# Relationship between bone turnover markers and the heel stiffness index measured by quantitative ultrasound in middle-aged and elderly Japanese men

**DOI:** 10.1097/MD.0000000000009962

**Published:** 2018-02-23

**Authors:** Takayuki Nishimura, Kazuhiko Arima, Yasuyo Abe, Mitsuo Kanagae, Satoshi Mizukami, Takuhiro Okabe, Yoshihito Tomita, Hisashi Goto, Itsuko Horiguchi, Kiyoshi Aoyagi

**Affiliations:** a Department of Public Health, Nagasaki University Graduate School of Biomedical Sciences, Sakamoto; b Department of Rehabilitation, Nishi-Isahaya Hospital, Kaizumachi; c Ken-Hoku Health Care Office, Tabiramachi; d Center for Public Relations Strategy, Nagasaki University, Bunkyoumachi, Nagasaki, Japan.

**Keywords:** bone turnover markers, elderly, men, osteoporosis, quantitative ultrasound

## Abstract

The aim of the present study was to investigate the age-related patterns and the relationships between serum levels of tartrate-resistant acid phosphatase-5b (TRACP-5b) or bone-specific alkaline phosphatase (BAP), and the heel stiffness index measured by quantitative ultrasound (QUS) in 429 Japanese men, with special emphasis on 2 age groups (40–59 years and 60 years or over). The heel stiffness index (bone mass) was measured by QUS. Serum samples were collected, and TRACP-5b and BAP levels were measured. The stiffness index was significantly decreased with age. Log (TRACP-5b) was significantly increased with age, but Log (BAP) was stable. Generalized linear models showed that higher levels of Log (TRACP-5b) and Log (BAP) were correlated with a lower stiffness index after adjusting for covariates in men aged 60 years or over, but not in men aged 40 to 59 years. In conclusion, higher rates of bone turnover markers were associated with a lower stiffness index only in elderly men. These results may indicate a different mechanism of low bone mass among different age groups of men.

## 1 Introduction

Osteoporosis is a systemic disease caused by low bone mass and micro-architectural deterioration of bone tissue, and resulting fractures impair activities of daily living and quality of life, leading to increased morbidity and mortality in the elderly.^[1,2]^ Although osteoporosis is more common in women, men have substantial age-related decreases in bone mineral density (BMD).^[3–5]^ Thus, osteoporosis in men is also a significant public health problem in this rapidly aging society.

Biochemical markers have been developed for the estimation of the rate of bone turnover and evaluate bone resorption or formation. Tartrate-resistant acid phosphatase-5b (TRACP-5b) is 1 of the bone resorption markers. Its concentration in the serum is determined by the number and activity of the osteoclasts involved in the resorption process.^[6]^ Previous studies showed that the serum TRACP-5b increased after menopause in women.^[7–10]^ However, age-related patterns of TRACP-5b in men have been limited; 1 study reported that TRACP-5b increased with age,^[7]^ but another reported that TRACP-5b was stable.^[8]^ On the contrary, bone-specific alkaline phosphatase (BAP) is on1 of the biochemical markers of bone formation. BAP is secreted by osteoblasts, and its increased concentration in the serum indicates primarily increased osteoblast activity or secondarily a repair reaction as a result of increased bone resorption.^[11]^ Previous studies showed that the serum BAP increased after menopause in women,^[12,13]^ but that BAP was stable with age in men.^[13–17]^

There have been many studies of the relationships between biochemical markers of bone turnover and BMD in women,^[7,8,10,12,13]^ but few in men.^[7,8]^ Only 2 studies showed that TRACP-5b correlated inversely and significantly with total BMD and BMDs of some skeletal sites in men.^[7,8]^ Some studies reported that BAP correlated inversely and significantly with BMD,^[15–17]^ but another reported no significant correlation in men.^[18]^ Furthermore, the evidence on the relationship between bone turnover markers and bone mass measured by quantitative ultrasound (QUS) measurement is limited in both men^[19,20]^ and women.^[9,21,22]^ QUS is inexpensive and noninvasive measure of bone, and is a strong predictor of osteoporotic fracture as BMD.^[23,24]^

Szulc et al^[17]^ reported that bone resorption markers, but not bone formation markers, increase and are associated with lower BMD after the age of 60 years, suggesting that this imbalance is responsible for increasing bone loss in elderly men. They also argued that levels of biochemical bone markers were very high in young men and decreased rapidly until the age of 40 years, and then more slowly until 60 years of age; after the age of 60 years, markers of bone formation remained stable, whereas resorption markers showed a moderate and variable increase with aging,^[17]^ which suggested that levels of biochemical bone markers change after age of 60 years.

We recently reported a significant relationship between urinary cross-linked N-telopeptide of type-I collagen (bone resorption marker) and the heel stiffness index measured by QUS only in men aged 60 years or over, but not in men aged 40 to 59 years, suggesting the different mechanisms for a low stiffness index among different age group.^[25]^ To overcome a limitation that at our previous study there were no bone formation markers available, we conducted this study including the measures both of bone formation markers and bone resorption markers. To the best of our knowledge, no study has examined relationships between TRACP-5b or BAP, and bone mass measured by QUS in men belonging to different age groups.

Therefore, the aim of the present study was to investigate the age-related patterns of serum TRACP-5b and BAP levels, and to determine their associations with the heel stiffness index measured by QUS in men with special reference to 2 specific age groups (40–59 years, and 60 years or over).

## 2 Methods

The subjects were community-dwelling men aged 40 years and over in Unzen city, Nagasaki Prefecture, Japan (target population approximately 13,000), who were invited to participate in periodic health examinations in 2011 to 2013 (the Unzen study). In all, 441 men (mean [SD] age: 66.1 [9.9], range 40–92 years) participated in this study. All subjects gave their written informed consent before examination. This study was approved by the Ethics Committee of Nagasaki University Graduate School of Biomedical Sciences.

The heel stiffness index (bone mass) by QUS was measured using a Lunar Achilles device (GE Lunar Corp., Madison, WI). Spot blood samples were collected. Serum TRACP-5b was measured by enzyme immunoassay and serum BAP was measured by chemiluminescence enzyme immunoassay. Height (m) and weight (kg) were measured with light clothing and without shoes, and the body mass index (BMI) was calculated as weight/height squared (kg/m^2^). Grip strength was measured using a hydraulic hand dynamometer (Jamar hydraulic hand dynamometer; Jafayette Instrument Company, Inc., Jafayette, IN). The average performance from 2 trials used their dominant hand was deemed as the result. Information on current smoking (yes/no) and alcohol drinking (≥40 g/d) was collected by interview.

Men with missing values for any variables were excluded from analysis (n = 12), leaving 429 men for the final data analysis. Since TRACP-5b and BAP were not normally distributed, these markers were treated as Log (TRACP-5b) and Log (BAP). Student *t* test or the chi-square test used to examine the differences in variables between 2 age groups (40–59 years, ≥60 years). One-way analysis of variance (ANOVA) was used to compare TRACP-5b and BAP among 10-year age groups. Generalized linear models were used to assess correlations between the stiffness index and TRACP-5b or BAP after adjusting for covariates (age, BMI, grip strength, current smoking, and alcohol drinking). A *P* value of less than .05 was considered significant. The data were analyzed using the Statistical Analysis System software package version 9.4 (SAS Institute, Cary, NC).

## 3 Results

Table 1 summarizes the characteristics of the 429 subjects. Weight, height, and BMI were significantly less in men aged 60 years or over than in men aged 40 to 59 years (*P* < .001 for weight and height, *P* = .014 for BMI). The stiffness index and grip strength were significantly lower in men aged 60 years or over than in men aged 40 to 59 years (*P* < .001). Log (TRACP-5b) and Log (BAP) were significantly higher in men aged 60 years or over than in men aged 40 to 59 years (*P* < .001 and *P* = .027, respectively). Prevalence of current smoking was significantly lower in men aged 60 years or over than in men aged 40 to 59 years (*P* < .001), but that of alcohol drinking was not significant (*P* = .078).

**Table 1:**
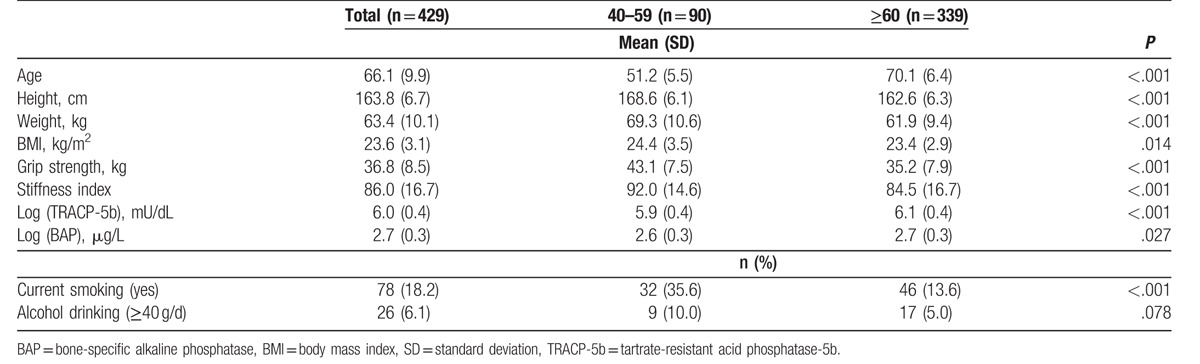
Characteristics of the study population (n = 429).

On 1-way ANOVA, the stiffness index decreased and Log (TRACP-5b) increased with age (*P* < .001), but there was no significant difference in Log (BAP) (*P* = .129) (Table 2).

**Table 2:**
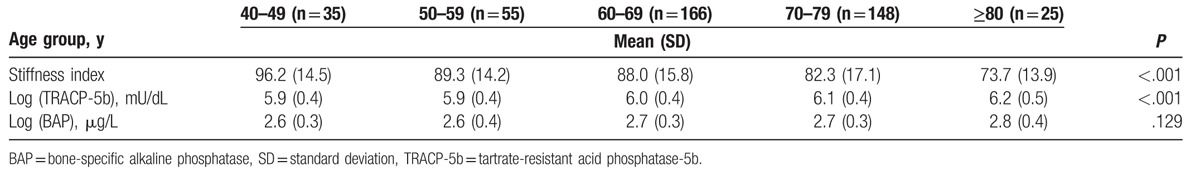
Mean (SD) of stiffness index, TRACP-5b, and BAP by age groups.

In men aged 60 years or over, generalized linear model showed that a higher level of serum Log (TRACP-5b) was significantly correlated with a lower stiffness index (QUS) (Table 3). Similarly, a higher level of serum Log (BAP) marginally correlated with a lower stiffness index in men aged 60 years or over (*P* = .055; Table 4). However, in men aged 40 to 59 years, there were no significant correlations between bone turnover markers and the stiffness index (Tables 3 and 4).

**Table 3:**
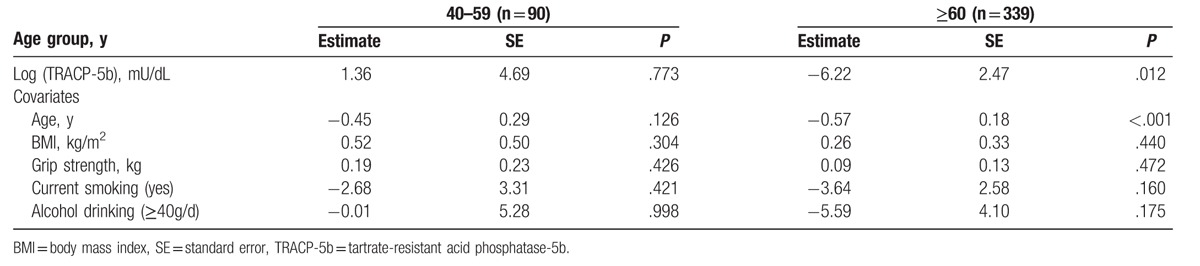
Generalized linear model between stiffness index and TRACP-5b.

**Table 4:**
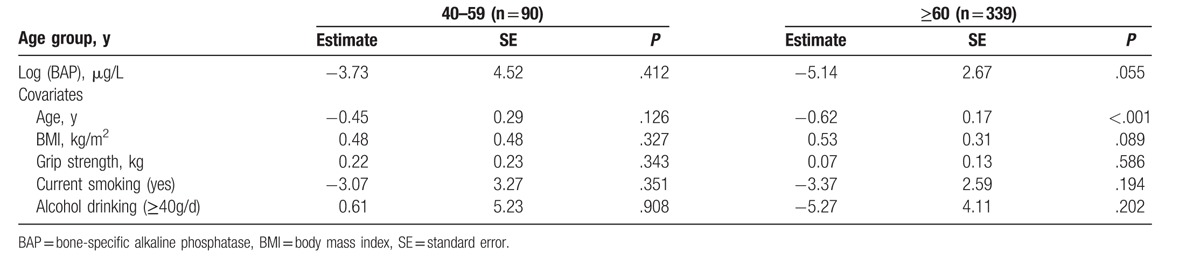
Generalized linear model between stiffness index and BAP.

We also analyzed the interaction between age and bone turnover markers with respect to stiffness index among all subjects in generalized linear model. The interaction between age and Log (TRACP-5b) with respect to stiffness index was significant (*P* = .04), but that between age and Log (BAP) was not. In the analysis of two age groups (aged 40–59 years, and 60 years or over), there was no significant interaction between age and bone turnover markers (Log (TRACP-5b) or Log (BAP)).

## 4 Discussion

A limited number of association were reported between various bone turnover markers and BMD,^[7,8,15–17]^ or the stiffness index (QUS)^[19,20]^ in men, previously. So far, 1 study reported relationships between TRACP-5b or BAP, and bone mass measured by QUS in women,^[9]^ but there has been no study in men. In the present study, we selected TRACP-5b and BAP from various bone turnover markers. This is the first study to show the relationships of TRACP-5b and BAP with the stiffness index (QUS) in men.

In the present study, TRACP-5b was significantly increased with age in men. Indridason et al^[7]^ reported that TRACP-5b increased moderately with age, which was consistent with the present result. In addition, there was no significant difference in BAP among 10-year age groups, which is consistent with previous studies.^[13–17]^ However, the evidence is still insufficient. Further study is needed to explore the age-related patterns of TRACP-5b and BAP in men.

Previous studies showed that TRACP-5b was significantly correlated with BMD in men,^[7,8]^ but these studies were conducted in a combined age groups (young, middle-aged, and elderly).^[7,8]^ In the present study, we analyzed with special reference to 2 specific age groups; a higher level of TRACP-5b was significantly correlated with a lower stiffness index (QUS) after adjusting for covariates in men aged 60 years or over, but not in men aged 40 to 59 years. The interaction between age and Log (TRACP-5b) with respect to stiffness index was significant among all subjects, but this significance disappeared in different age groups (aged 40–59 years, and 60 years or over). This result suggested that the effect of TRACP-5b on stiffness index was different between men aged 40 to 59 years and 60 years or over. These findings suggest that higher rates of bone resorption are associated with lower bone mass, especially in elderly men, but not in middle-aged men.

In the present study, a higher level of BAP was marginally correlated with a lower stiffness index after adjusting for covariates in men aged 60 years or over, but not in men aged 40 to 59 years. Previous studies reported a weak inverse relationship between BAP and BMDs of some skeletal sites in men.^[15–17]^ Additionally, Khosla et al^[16]^ and Szulc et al^[17]^ reported that this inverse relationship is clearer in elderly men. These results suggest that higher rates of bone formation are associated with lower bone mass, especially in elderly men. However, there was a possibility not to reach the statistical significance because of relatively small sample size in aged 40 to 59 years. We need further studies to clarify this point.

In women, bone resorption markers and formation markers increase in parallel after menopause,^[7–10,12,13]^ which indicates high bone turnover and causes bone loss. In men, however, previous studies demonstrated that increases of bone resorption markers and formation markers did not occur in parallel.^[14,15,17]^ Furthermore, in elderly men, slightly increased bone resorption is not matched by a parallel increase in bone formation; this imbalance results in the age-related bone loss.^[14,17]^

The present study has several limitations. First, since this study was cross-sectional in design, and the results do not necessarily show a causal relationship. Second, information on other determinants (eg, genetic background or nutritional status) contributing to skeletal maintenance in aging men was not available. Finally, the subjects were participants in a health examination and may not be representative of the general population.

## 5 Conclusions

In conclusion, higher levels of TRACP-5b and BAP were correlated with a lower stiffness index (QUS) in men aged 60 years or over, but not in men aged 40 to 59 years. Higher rates of bone resorption and formation were associated with a lower stiffness index only in elderly men. The present results may indicate different mechanisms for a low stiffness index among different age groups.
